# Cytokine alterations in pediatric internalizing disorders: Systematic review and exploratory multi-variate meta-analysis

**DOI:** 10.1016/j.bbih.2022.100490

**Published:** 2022-07-16

**Authors:** Aaron S. Howe, David A. Lynch

**Affiliations:** a Department of Clinical & Counseling Psychology, Teacher's College, Columbia University, 525 West 120th Street, NY, NY, 10027, USA; b Department of Psychiatry, Columbia University - Vagelos College of Physicians and Surgeons, Columbia University Irving Medical Center, and New York-Presbyterian, 5 Columbus Circle, New York, NY, 10019, USA

**Keywords:** Cytokine, Internalizing disorders, Inflammation, Child, Adolescent

## Abstract

Pediatric internalizing disorders are prevalent and characterized by a maladaptive cognitive, emotional response to a perceived stressor. The hypothesized effect of this response is observable changes in behavior mediated by homeostatic inflammatory cytokines. The aim of this study was to synthesize the literature and analyze the effect of cytokines on pediatric internalizing disorders. Influential moderating variables, including mean body mass index, fasting status at blood collection, participant sex, cytokine type, mean age, percentage of sample medicated, and diagnosis, were also assessed. A systematic literature search was performed in electronic databases (Medline, PubMed, and PsycINFO) from January 1, 1980 to June 15, 2022. Case-control studies of pediatric internalizing disorders, specifically anxiety and depression, were reviewed for their association with peripheral cytokine levels. Meta-analyses were performed using a random effects multi-variate model and effect sizes were calculated using Hedge's *g* for IL-2, CRP, IL-6, TNF-α, IL-1β, IFN-γ, and IL-10. Thirty-three studies were reviewed and 28 studies were included in the meta-analysis (*n* = 1322 cases and *n* = 3617 controls). Peripheral cytokine levels were elevated in pediatric internalizing disorders compared to controls (Hedge's *g* = 0.19, *p* < 0.001). In the moderator analyses, depression diagnosis (Hedge's *g* = 0.18, *p* = 0.009) and non-fasting blood collection (Hedge's *g* = 0.20, *p* = 0.006) were significant. The meta-analytic findings are limited by methodological variation between studies, high heterogeneity, and low statistical power. Despite this, the findings suggest that elevated peripheral cytokine levels may play a role in the etiology and/or symptom maintenance of pediatric internalizing disorders.

## Highlights

• Depression diagnosis and non-fasting blood collection were observed to significantly influenced peripheral cytokine levels.
• No individual peripheral cytokines tested were associated with pediatric internalizing disorders.
• Overall, elevated peripheral cytokines were observed in pediatric internalizing disorders compared to controls.

## 1 Introduction

Internalizing disorders are primarily characterized by anxiety, depressive and somatization symptoms that are experienced because of a maladaptive cognitive, emotional schema within the self as a response to a stressor (APA, 2022; De Baumont et al., 2019). These disorders pose a significant public health concern due to their early age at onset, increasing prevalence, and burden on the health care system (Cummings et al., 2014). The onset of internalizing disorders is early to middle childhood (6–11 years old) for anxiety and middle to late adolescence (15–19 years old) for depression; however, sub-threshold symptoms often precede a formal diagnosis (Costello et al., 2003; Kessler et al., 2005). A study analyzing a national US survey of adolescents aged 13–18 determined lifetime prevalence rates of a DSM-IV anxiety and depressive diagnosis was 31.9% and 15.9%, respectively (Merikangas et al., 2010). These disorders are often co-morbid creating substantial functional impairment (e.g., reduced social behaviors, interpersonal problems, academic difficulties), disruptive familial discord, and increasing risk of suicidal behavior with age (Cummings et al., 2014; Jha et al., 2020; Moriarity et al., 2018).

Historically, research on internalizing disorders has focused on the interaction or deterministic contributions of genetic and environmental factors (Haroon et al., 2012; Miller & Cole, 2012). These studies have facilitated a broader scope on the etiology of internalizing disorders to incorporate a multi-system model that considers the coordinated impact of several biological mechanisms, organized by neural pathways involved in immune, endocrine, and epigenetic responses to internal and external stressors (Flouri et al., 2019; Miller et al., 2009). Given the transdiagnostic symptoms, shared etiological pathways, and high rates of co-morbidity within these disorders, current research has focused on identifying biological commonalities that may better predict the relationship between the brain and psychopathological behavior (Miller and Chen, 2012). As such, systemic inflammation has become an emerging mechanism of interest as a shared pathway of biological pathology in this heterogenous group of disorders (Haroon et al., 2012). Studies have shown a relationship between inflammation with acute (e.g., bacterial, and viral infection) and chronic physical (e.g., cardiovascular disease, diabetes, metabolic syndrome) and psychological conditions (e.g., psychosis, attention-deficit/hyperactivity disorder, eating disorders) (Mills et al., 2013; Flouri et al., 2020). Increased levels of inflammation have been associated with “sickness” symptoms that overlap with that of internalizing disorders, which include anhedonia, low mood, fatigue, sleep disturbance, social and behavioral withdrawal (Freed et al., 2019; Giollabhui et al., 2019). This inflammatory phenotype is thought to be mediated by cytokines that commence the acute inflammatory response and maintain chronic, perpetual positive feedback loops with the stress regulation systems of the sympathetic nervous system and hypothalamic-pituitary-adrenal (HPA) axis (Miller and Cole, 2012).

Cytokines are immunomodulating proteins that are involved in cell signaling of the initiation (termed “the acute phase response”) and maintenance of immune responses (Peters et al., 2021). They regulate communication between immune system cells and other tissues via cell-specific receptor activation to produce a wide-spread, inflammatory response that alters neuro-chemical and neuro-endocrine processes, which subsequently modulates behavior (Miller and Cole, 2012). They have the unique ability to travel throughout the body via several different mechanisms: a) passive transport through selective endothelial permeability of the blood-brain-barrier, b) active transport via soluble transporter proteins, c) activation of endothelial cells, d) direct receptor binding to peripheral afferent nerve fibres, and e) migration through the lymphatic system to target organ systems (Miller and Cole, 2012). These mechanisms of transport allow for cytokines to perform pleiotropic actions throughout the body in response to acute, acute-repeated, and chronic stressors. Cytokines are produced primarily by macrophages that can be recruited to the central nervous system (CNS) or produced within by microglia, resident macrophages, and dendritic cells (Slavich et al., 2014). In the CNS, cytokines influence degradation, release, and re-uptake of neurotransmitters (e.g., serotonin, norepinephrine, and dopamine) (Gabbay et al., 2010; Michels et al., 2018; Oztürk et al., 2020), autoimmunity and production of antibodies (Himmerich et al., 2019), regulation and stimulation of the HPA axis (Mills et al., 2013), stimulation and inhibition of vagal nerve tone (Toenders et al., 2022), and promotion or suppression of neuroplasticity (e.g., neurogenesis, synaptic re-modeling) (Miller et al., 2009).

The “*Macrophage/Cytokine Theory of Depression*” was the first theory to postulate that cytokines could be involved in the etiology of depression (Smith, 1991; Ur et al., 1992). This theory states that excessive cytokine activity is associated with monoamine neurotransmission dysfunction and profound endocrine abnormalities, which are commonly reported associations in studies of internalizing disorders (Mills et al., 2013; Peters et al., 2019). This is further supported by increased prevalence rates of depression in females (Byrne et al., 2015; Merikangas et al., 2010) and cytokine treatment studies of IFN-α (interferon-alpha) in cancer patients, where it was observed that higher doses of cytokine therapies had produced increased depression symptoms (Capuron et al., 2002; Musselman et al., 2001). Therefore, it has been hypothesized that cytokines may be mediating the relationship between inflammation and internalizing disorders through perpetuation of chronic, low-grade inflammation (Schleifer et al., 2002) or induction of dysfunctional positive feedback loops within the stress regulation systems of the body (Amitai et al., 2016; Lee et al., 2020).

Recent research on cytokines has largely focused on major depression and anxiety disorders in adult populations. Meta-analyses have identified robust associations between elevations of pro-inflammatory cytokines in major depression (TNF-α and IL-6) (Dowlati et al., 2010; Köhler et al., 2017) and anxiety disorders (IL-6, TNF-α, and IL-1β) (Renna et al., 2018). In pediatric samples, the association between cytokines and internalizing disorders remain elusive and conflicting. Longitudinal studies of both community and clinical pediatric samples have observed a bi-directional association between internalizing symptoms and peripheral pro-inflammatory cytokines, IL-6 and TNF-α (De Baumont et al., 2019; Flouri et al., 2019, 2020; Kautz et al., 2020; Khandaker et al., 2018; Miller et al., 2009; Miller and Cole, 2012; Pearlstein et al., 2020; Rengasamy et al., 2021a). However, in some anti-depressant treatment studies, pro-inflammatory cytokine levels appear to normalize to baseline or remain elevated despite reductions in internalizing symptoms over time in depressed and anxious youth (Amitai et al., 2016; Becerril-Villanueva et al., 2019; Pérez-Sánchez et al., 2018). A systematic review and meta-analysis of peripheral pro-inflammatory cytokines in pediatric depression identified eight studies for qualitative synthesis and five studies for meta-analysis (D’Acunto et al., 2019). This meta-analysis observed a marginal association between TNF-α and depressive disorders in children and adolescents; however, the statistical power was considerably low compared to analyses conducted in adults. Moderator analyses were also not performed on the data and therefore, it is unclear whether confounders related to immune system alterations (e.g., changes in hormonal inputs and immune system organ size) or changes in communication between the brain-gut-immune axis (e.g., synaptic pruning/plasticity, adiposity, immune cell responsivity) that occur during youth development contributed to false negative findings (Himmerich et al., 2019; Toenders et al., 2022). A second meta-analysis focused on pediatric depression including both cross-sectional and longitudinal studies found a positive association with concurrent and future inflammation, but not peripheral cytokine levels specifically (Colasanto et al., 2020). Given the low statistical power and lack of investigation of moderating variables in previous analyses, it remains unknown if there is an associative relationship between peripheral cytokine levels and internalizing disorders in pediatric samples.

The primary aim of this review and meta-analysis was to integrate the extant literature to evaluate the evidence for an association between peripheral cytokine levels and pediatric internalizing disorders. It is hypothesized that those with pediatric internalizing disorders will present with elevations in peripheral cytokines consistent with findings reported in adult samples (Dowlati et al., 2010; Köhler et al., 2017; Renna et al., 2018). A secondary aim of this review is to investigate study heterogeneity through moderator analyses of cytokine type and influential variables (i.e., mean age, medication, mean body mass index, fasting status at blood collection, participant sex, and diagnosis) identified in the literature that may influence peripheral cytokine levels in pediatric studies. Older age in child development is associated with intrinsic changes in hormone activity related to pubertal status and maturation of immune system organs (Stumper et al., 2020). Theses age-related changes have been associated with fluctuating changes in pro-inflammatory cytokine levels for pediatric depression and anxiety longitudinally (De Baumont et al., 2019; Perry et al., 2020). Adiposity, commonly measured via body mass index (BMI), has been shown to be independently associated with decreases in basal TNF-α levels (Dixon et al., 2009) and increases in IL-6 in response to psychosocial stress in healthy pediatric samples (McInnis et al., 2014). Differential cytokine levels have been reported between pediatric females and males with females reporting greater variability (Walss-Bass et al., 2018) and larger differences in case-control studies of depression (Pallavi et al., 2015). Anti-depressant treatment has been shown to modulate pro-inflammatory cytokine levels after short-term administration in first-episode pediatric depression (Lee et al., 2020) and recurrent pediatric depression (Amitai et al., 2016; -Pérez-Sánchez et al., 2018). Intermittent and single-day fasting has also been shown to alter peripheral cytokine levels in healthy samples (Aksungar et al., 2007; Faris et al., 2012). Lastly, depressive diagnoses compared to anxiety diagnoses have been shown to report a more consistent association with elevations in peripheral cytokine levels in adult studies (Dowlati et al., 2010; Renna et al., 2018). No a priori hypotheses were formulated for these variables as these analyses were considered exploratory.

## 2 Materials and methods

### 2.1 Search strategy

**Fig. 1:**
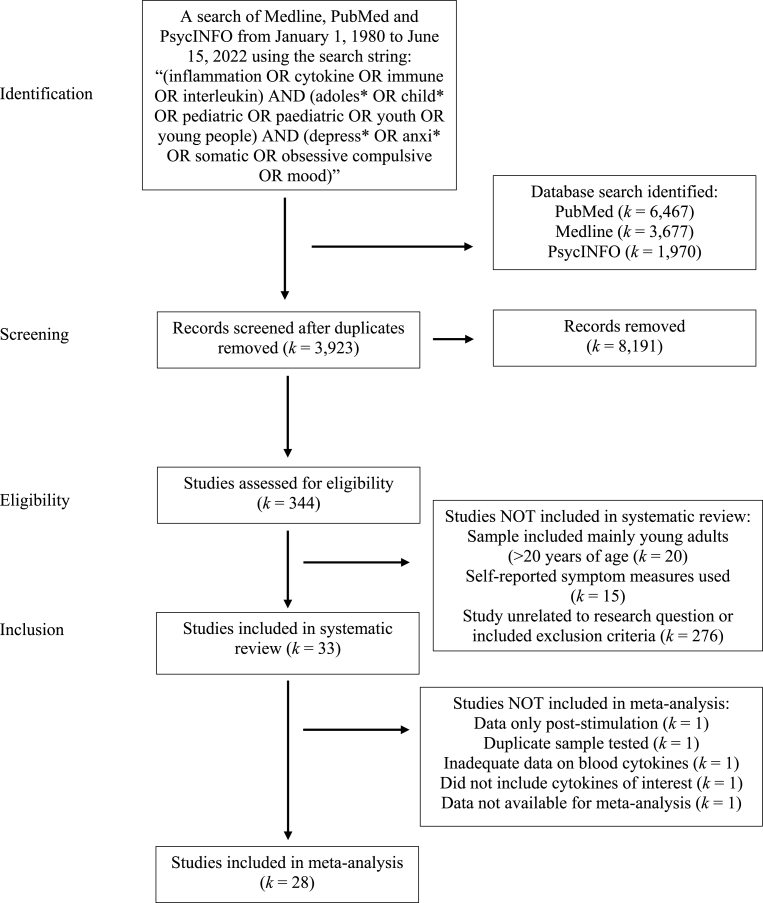
A PRISMA summary of the search strategy and outcome.

This systematic review was undertaken and reported in accordance with the PRISMA 2020 guidelines (Preferred Reporting Items for Systematic Reviews and Meta-Analysis; Page et al., 2021). A computerized search of Medline, PubMed and PsycINFO from January 1, 1980 to June 15, 2022 was conducted using the search strategy “(inflammation OR cytokine OR immune OR interleukin) AND (adoles* OR child* OR pediatric OR paediatric OR youth OR young people) AND (depress* OR anxi* OR somatic OR obsessive compulsive OR mood).” The search outcome was reviewed by both authors. Studies were filtered by Human species and English language only. The primary purpose of the search was to identify all studies that reported empirical data on cytokine measurement in pediatric internalizing disorders within a case-control design. Duplicates were deleted from the search and abstracts of each article was scanned for relevance to the review. The full text of selected studies was then retrieved independently and assessed for eligibility with the established criteria collaboratively. The reference list(s) of the selected studies and published reviews were also screened and considered for inclusion. Authors were contacted for empirical data in studies that did not report cytokine measurement within their published work or supplementary materials. A summary of the search outcome is illustrated in Fig. 1.

### 2.2 Inclusion and exclusion criteria

Studies comparing peripheral cytokines levels in pediatric internalizing disorders, including individuals diagnosed with depression, somatic, post-traumatic stress disorder, and anxiety-based disorders, and controls were included in the systematic review and meta-analysis. We decided to combine these disorders based on shared universal dimensions of negative affectivity, clinical overlap in symptom presentation, and empirical evidence for cytokine dysregulation (Merikangas et al., 2010; Renna et al., 2018; Himmerich et al., 2019). The inclusion criteria is described as follows: (1) included a pediatric sample comprising of pre-dominantly children and adolescents aged 19 or younger (World Health Organization, 2021); (2) study participants were diagnosed with somatization, depression, post-traumatic stress disorder or anxiety according to standardized clinician administered instruments using validated criteria as outlined in ICD and/or DSM criteria (3) cross-sectional or longitudinal (e.g., treatment studies, birth cohort studies); case-control study design where baseline, untreated cytokine measurements were reported or provided upon request; (4) measurement of peripheral cytokine levels from blood plasma or serum samples; (5) studies which provided sufficient quantitative data (e.g., mean, standard deviation, *p*-values) to calculate an effect size. Studies were excluded if: (1) cytokines were measured in cerebrospinal fluid, brain tissue, tissue culture, or saliva samples; (2) cytokines were measured only after stimulation with lipopolysaccharide or another immune inducing agent; (3) measurement of immune markers other than cytokines (e.g., active phase proteins, chemokines). Studies that reported co-morbidities were included insofar that the co-morbidities were affective or behavioral disorders.

### 2.3 Data extraction

Demographic variables, methodological variables, and cytokine measurements were extracted from the studies. Demographic variables extracted included country of origin, mean age, percentage of females enrolled, mean body mass index (BMI) expressed as kg/m^2^, percentage of sample medicated, and participant sampling (coded as clinical or community). Primary diagnosis, co-morbid diagnoses, and diagnostic instruments used were also extracted and coded. Methodological variables extracted included time of blood sample collection, biological sample type (e.g., blood serum or plasma), immunological assay used, and intra-assay variability. All cytokines analyzed in the studies were coded and extracted separately for each study.

Most of the studies included means and standard deviations for the cytokines investigated. For studies that did not include means and standard deviations, authors of the individual studies were contacted via email (Byrne et al., 2013; Colak Sivri et al., 2018; De Baumont et al., 2019; Gariup et al., 2015; Gomes et al., 2021; Martins et al., 2021; Peters et al., 2019). The authors of some of these studies were able to provide any requested statistical data and provided demographic variables that were not reported in their published work (Byrne et al., 2013; Colak Sivri et al., 2018; De Baumont et al., 2019; Peters et al., 2019; Martins et al., 2021).

Case-control data was not available for Gomes et al., (2021) to be included in the meta-analysis. For Gariup et al., 2015 specifically, data retrieved was for individuals sub-grouped into anxiety, OCD, adjustment disorder and depression disorders, and data was excluded for psychosis and mania sub-groupings. Cytokines that were “not detected” or indicated that they were below the set immunoassay detection threshold were not included (Byrne et al., 2013; Gariup et al., 2015). Cytokine measurements for IL-6, IL-8 and IL-10 were not available for Reddy et al., (2020). There was one study that did not respond to contact and reported their statistics in medians and interquartile ranges (Pallavi et al., 2015). Data from this study was converted to an estimated mean and standard deviation using a precise, low bias statistical method under the assumption of a skewed raw dataset (Wan et al., 2014; Weir et al., 2018). A quality assessment of the studies included in the systematic review was performed and based on the criteria developed by Toenders et al. (2022) utilizing the Cochrane Consumers and Communication Review Group Study Quality Guide (2013) and the Critical Appraisal Skills Programme (CASP) (Ryan et al., 2013).

### 2.4 Statistical analysis

Analyses were performed using the “compute.es” version 0.2–5 (Del Re, 2013) and “metafor” version 3.0–2 (Viechtbauer, 2010) packages within R version 4.0.2 GUI 1.72 for Mac (Core Team R, 2021). Hedge's *g* effect sizes, including variances and 95% confidence intervals, were calculated for each cytokine measured between pediatric individuals diagnosed with internalizing disorders and controls within a given study. We used Hedge's *g* rather than other effect sizes as it includes a correction for small sample sizes (Van Den Noortgate and Onghena, 2003). The value of Hedge's *g* is > 0 when cytokines are elevated in pediatric internalizing disorders as compared to controls and < 0 if the opposite occurs. Effect sizes were interpreted using Cohen's (1988) guidelines of 0.2 = small, 0.5 = medium, and 0.8 = large. A multi-variate random-effects meta-analysis was performed with the calculated effect sizes considering cytokine and individual studies as nested, dependent variables. This approach was used to increase sample size and statistical power considering the small sample sizes of the individual studies included. We evaluated the sensitivity of our analyses through a comparative approach of fitted models with and without effect sizes that were identified as potential outliers, unmedicated studies only, and studies that had exclusively adolescent samples.

Heterogeneity between studies was assessed using *Q* and *I*^*2*^ statistic. A significant *Q* statistic indicates between study heterogeneity that could warrant exploration of moderating variables; however, it may be influenced by small sample size. The *I*^*2*^ statistic measures total variation in effect sizes due to study heterogeneity and is also influenced by sample size (Higgins et al., 2003). Estimated *I*^*2*^ was interpreted as <30% = low heterogeneity, ≥ 31% and ≤ 60% = moderate heterogeneity, and ≥ 61% = high heterogeneity. Due to data constraints and sample sizes required for model fitting, moderator analyses using a random-effects meta-regression model was performed for the following variables only: percentage of sample medicated, mean age, cytokine type, percentage of females enrolled (Oddy et al., 2018; Reid et al., 2020; Walss-Bass et al., 2018), mean BMI (Dixon et al., 2009; McInnis et al., 2014), fasting status at blood collection (Aksungar et al., 2007; Faris et al., 2012; Peters et al., 2019), and diagnosis (coded as either anxiety or depression). Percentage of females enrolled, mean age, mean BMI, and percentage of sample medicated were coded as continuous variables. Fasting status and diagnosis were coded as dichotomous variables. Studies that had a combined sample of both anxiety and depression were not included in the moderator analysis for diagnosis (Belem da Silva et al., 2017; Henje-Blom et al., 2012; Liu et al., 2021). We interpreted the moderator analysis as significant where the *p*-value was < 0.05. A forest plot was constructed to provide a visualization of the calculated effect sizes in pediatric individuals with internalizing disorders compared to controls. Publication bias was assessed by using Egger's regression test statistic and visual examination by funnel plot (Egger et al., 1997). For the Egger's regression test, if the intercept deviates from zero at a significance level of *p* = 0.1, we interpreted the analysis as biased (Egger et al., 1997).

## 3 Results

### 3.1 Search outcome

A summary of the 33 studies that examined cytokines in pediatric internalizing disorders and were included in this systematic review is provided in Table 1. There were 23 cross-sectional studies and 10 longitudinal studies (De Baumont et al., 2019; Ho et al., 2022; Pervanidou et al., 2007; Rengasamy et al., 2021b), of which four were pharmacological treatment studies (Becerril-Villanueva et al., 2019; Lee et al., 2020; Martins et al., 2021; Pérez-Sánchez et al., 2018) and two were birth cohort studies (Gomes et al., 2021; Perry et al., 2020). A total of 28 studies were included in the meta-analysis, which included 1322 (62.0% female) individuals with pediatric internalizing disorders and 3617 (55.6% female) controls. Within this subset of 28 studies, there were 18 studies on depression-based disorders, four studies on anxiety-based disorders, two studies on post-traumatic stress disorder, and four studies that had a combined sample of anxiety and depression. There were no studies identified in the literature search that investigated somatic disorders in pediatric samples.

**Table 1:** Summary of case-control studies investigating cytokines in internalizing disorders.

Study	Country	Participants	Age range	Diagnostic method	Internalizing disorder/co-morbidity	Cytokines investigated	Summary of findings
Brambilla et al., 2004*	Italy	Clinical (outpatient)	6-14y	K-SADS (DSM-IV)	MDD, DYS Co-morbidity: Axis I co-morbidities were excluded.	TNF-α, IL-1β	↑ IL-1β & TNF-α in MDD vs HC (NS). ↑ IL-1β in DYS vs HC (NS). ↓ TNF-α in DYS vs HC. No difference between MDD vs DYS. Negative correlation between depression severity and TNF-α in MDD patients. Positive correlation between depression and anxiety severity and IL-1β in MDD patients. Quality Rating: Moderate
Pervanidou et al., 2007*	Greece	Clinical (hospitalization)	7-18y	K-SADS-PL (DSM-IV)	PTSD-MVA Co-morbidity: Other mental disorders or pre-existing psychopathology were excluded.	IL-6	↑ morning IL-6 in PTSD > non-PTSD > HC at baseline, but not 1 month or 6 month follow up. Morning IL-6 positively correlated with PTSD severity at 6 month follow up. IL-6 levels correlated with salivary cortisol. Quality Rating: Moderate
Gabbay et al., 2009a*	USA	Clinical (inpatient/outpatient)	12-19y	K-SADS-PL (DSM-IV)	MDD Co-morbidity: n = 16 (53%) ADHD, Anxiety, ODD	IFN-γ, TNF-α, IL-6, IL-4, IL-1β	↑ IFN-γ in MDD vs HC. ↑ IFN-γ/IL-4 ratio in MDD vs HC. No correlations found for MDD features and the investigated cytokines. Quality Rating: High
Study	Country	Participants	Age range	Diagnostic method	Internalizing disorder/co-morbidity	Cytokines investigated	Summary of findings
Gabbay et al. (2009b)	USA	Clinical (inpatient/outpatient)	12-19y	K-SADS-PL (DSM-IV)	MDD Co-morbidity: n = 14 (78%) ADHD, Anxiety, ODD	IFN-γ, TNF-α, IL-6, IL-4, IL-1β	↓ TNF-α in suicidal MDD vs non-suicidal MDD. ↑ IFN-γ in suicidal MDD vs HC. ↑ IFN-γ in non-suicidal MDD vs HC. ↑ IFN-γ/IL-4 ratio in non-suicidal MDD vs HC. Quality Rating: Moderate
Henje-Blom et al., 2012*	Sweden	Clinical (outpatient)	14-18y	DAWBA (DSM-IV)	GAD, SoP, SP, PD, SAD, PTSD and/or MDD Comorbidity: permitted, but details not reported	IFN-γ, TNF-α, IL-2, IL-6, IL-1β, IL-10	↑ IL-2, IL-1β, IL-10 in clinical sample vs HC. ↑ IL-2 in medicated clinical sample vs HC. ↑ IL-6 in unmedicated clinical sample vs HC. ↑ IFN-γ, IL-6 associated with self-assessment of more severe depression and anxiety symptoms. Quality Rating: Moderate
Byrne et al. (2013)	Australia	Community (high school)	12-18y	K-SADS (DSM-IV)	MDD, DNOS Co-morbidity: Not specified	IL-2, IL-4, IL-6, IL-8, IL-1α, IL-10, IL-12, IL-13 IL-17, TNF-α, IFN-α2, IFN-γ	No difference in the investigated cytokines between depressed and healthy adolescents. Quality Rating: Moderate
Gariup et al., 2015*	Spain	Clinical (inpatient)	8-17y	K-SADS-PL (DSM-IV-TR)	Anxiety, Depression Co-morbidity: Not specified	IL-1β, IL-2, IL-4, IL-5, IL-6, IL-8, IL-10, IFN-γ, TNF-α, GM-CSF	↑ IL-1β in anxiety and depression vs HC. ↑ IL-8 in anxiety and depression vs HC. ↑ IL-6 and IL-10 in depression. Quality Rating: Moderate
Study	Country	Participants	Age range	Diagnostic method	Internalizing disorder/co-morbidity	Cytokines investigated	Summary of findings
Pallavi et al., 2015*	India	Clinical (outpatient)	13-18y	SCID-I/NP (DSM-IV)	Depression Co-morbidity: Not specified	IL-1β, IL-2, IL-6, TGF-β1 IFN-γ, IL-17, TNF-α, IL-10	↑ IL-2 and IL-6 in depression vs HC. ↑ IL-2 in male depression vs male HC. ↑ IL-6 in female depression vs female HC. ↑ TGF-β1 in medicated vs non-medicated depression. Negative correlation between depression, state anxiety, trait anxiety scores and TGF-β1 levels. In depressed females, TNF-α positively correlated with depression scores. Quality Rating: Moderate
Ayaydin et al., 2016*	Turkey	Clinical (outpatient)	13-18y	K-SADS-PL (DSM-IV)	PTSD Co-morbidity: MDD (61%)	IFN-γ, TNF-α	↓ IFN-γ in PTSD vs HC. Quality Rating: Moderate
Miklowitz et al., 2016*	USA	clinical (outpatient)	12-18y	K-SADS-PL (DSM-IV-TR)	MDD Co-morbidity: ADHD (30.8%) Anxiety (38.5%)	IL-1β, IL-6, IL-8, IL-10, TNF-α	No difference in tested cytokines between MDD vs HC. No correlations with the tested cytokines and medication status or mood symptoms. Quality Rating: Moderate
Şimşek et al., 2016*	Turkey	clinical (hospitalization)	7-17y	K-SADS-PL (DSM-V)	OCD Co-morbidity: Tic Disorder (11.8%)	IL-2, IL-4, IL-6, IL-10, TNF-α, IFN-γ IL-17a	↑ IL-17a, TNF-α, and IL-2 in OCD vs HC. Negative correlation between TNF-α levels and OCD symptoms. Quality Rating: Moderate
Study	Country	Participants	Age range	Diagnostic method	Internalizing disorder/co-morbidity	Cytokines investigated	Summary of findings
Belem da Silva et al., 2017*	Brazil	Community (schools)	10-17y	K-SADS-PL (DSM-IV-TR)	MDD, GAD, SAD, SoP, PD Co-morbidity: Not specified	IL-6, IL-10	↑ IL-6 in internalizing disorders compared to those without internalizing disorders. Quality Rating: Moderate
Rodríguez et al., 2017*	Spain	Clinical (outpatient)	8-19y	K-SADS-PL (DSM-IV)	OCD Co-morbidity: Anxiety or mood disorder (45.1%) ADHD or tic disorder (21.6%)	IL-1β, IL-6, IL-8, TNF-α, GM-CSF	No difference in tested cytokines between OCD vs HC during basal conditions. Higher cytokine levels in OCD observed: LPS > DEX + LPS > basal conditions. After treatment with LPS, ↑ IL-1β, IL-6, IL-8, TNF-α, GM-CSF in OCD vs HC. After treatment with LPS + DEX, ↑ IL-1β, IL-6, and IL-8 in OCD vs HC. Quality Rating: Moderate
Colak-Sivri et al., 2018*	Turkey	Clinical (outpatient)	8-18y	K-SADS-PL (DSM-V)	OCD Co-morbidity: GAD (29.5%) SoP (27.3%) SAD (9.1%)	TNF-α, IL-17 TGF-β, IL-12,	↑ TNF-α in OCD vs HC. ↓ IL-12 in OCD vs HC. No association between OCD symptoms and any of the tested cytokines. Quality Rating: Moderate
Pérez-Sánchez et al., 2018*	Mexico	Clinical (outpatient)	14-19y	MINI v.5 (DSM-IV-TR)	MDD Co-morbidity: Not specified	IL-2, IL-12p70, IFN-γ, IL-1β, TNF-α, IL-6, IL-15, IL-4, IL-5, IL-13, IL-1Ra, IL-10	↑ IL-2, IFN-γ, TNF-α, IL-1β, IL-6, IL-12p70, and IL-15 in MDD vs HC. ↑ IL-2 in MDD after 8 weeks of treatment with anti-depressant, fluoxetine. ↓ IFN-γ, TNF-α, IL-1β, IL-6, IL-12p70, and IL-15 in MDD after 4 weeks of treatment with anti-depressant, fluoxetine, however levels restored after 8 weeks. Quality Rating: Moderate
Study	Country	Participants	Age range	Diagnostic Method	Internalizing Disorder/Co-morbidity	Cytokines investigated	Summary of findings
Petrov et al., 2018*	USA	Clinical (outpatient)	6-13y	K-SADS-PL (DSM-V)	MDD Co-morbidity: Not specified	IL-6	Significant positive association between BMI and IL-6 for both non-mood and mood disorders. No significant difference in IL-6 levels between MDD vs HC and MDD vs BD contrasts. Quality Rating: Moderate
Becerril-Villanueva et al. (2019)	Mexico	Clinical (outpatient)	14-19y	MINI v.5 (DSM-IV-TR)	MDD Co-morbidity: Not specified	IL-7, IL-9, IL-17a, VEGF, FGF, G-CSF, GM-CSF	↑ IL-7, IL-9, IL-17a and VEGF in MDD vs HC. ↑ FGF, G-CSF and GM-CSF in MDD vs HC. ↓ FGF, G-CSF and GM-CSF in MDD after 4 weeks of treatment with anti-depressant, fluoxetine, however levels restored after 8 weeks. No correlation between depression severity and tested cytokines. Quality Rating: Moderate
Calarge et al., 2019*	USA	Clinical (outpatient)	12-17y	MINI v.6 (DSM-V)	MDD Comorbidity: permitted, but details not reported	IL-6, IL-1β, TNF-α	No significant difference between MDD vs HC for all cytokines tested. Depression severity was positively correlated with TNF-α. Neuro-vegetative symptom severity was positively correlated with IL-1β. Quality Rating: Moderate
Study	Country	Participants	Age range	Diagnostic method	Internalizing disorder/co-morbidity	Cytokines investigated	Summary of findings
De Baumont et al., 2019*	Brazil	Community (schools)	8-18y	K-SADS-PL (DSM-IV)	GAD, SoP, SP, SAD, PD Co-morbidity: Not specified	IL-6, IL-10, IL-1β, TNF-α	↑ IL-6 for anxiety diagnosis vs HC at 5 years follow up, but not baseline. ↑ HDL-cholesterol partially mediated the relationship between anxiety diagnosis and IL-6. Quality Rating: Moderate
Freed et al. (2019)	USA	Community (not specified)	12-20y	K-SADS-PL (DSM-IV)	MDD, Anxiety Co-morbidity: Not specified	EGF, MCP-3, Eotaxin, FGF-2. Flt3-L, VEGF Fractalkine, G-CSF, GM-CSF, GRO, IFN-α2, IL-2 IFN-γ, IL-1α, IL-1β, IL-1ra, IL-3, TGF-α, IL-5, PDGF, IL-9, IL-10, MDC IL-12p40, IL-7, IL-12p70, IL-8, IL-13, IL-15, IP-10, MCP-1, IL-6 IL-17a, IL-4 MIP-1α, MIP-1β, TNF-α, TNF- β, sCD40L, RANTES	No difference in tested cytokines between adolescents with psychiatric disorders vs HC after LPS administration. Anhedonia score positively correlated with FGF-2, Flt3-L, Fractalkine, G-CSF, IL-1α, IL-2, IL-3, IL-4, IL-7, IL-9, IL-10, IL-12p40, IL-12p70, IL-15, IL-17α, TNF-β, VEGF in the psychiatric sample. Quality Rating: High
Study	Country	Participants	Age range	Diagnostic method	Internalizing disorder/co-morbidity	Cytokines investigated	Summary of findings
Peters et al., 2019*	USA	Clinical (outpatient) and Community	12-17y	K-SADS-PL (DSM-IV)	Depression Co-morbidity: PD (10.0%) SP (2.5%) SoP (27.5%) AG (2.5%) GAD (42.5%) PTSD (12.5%) ADHD (15.0%) ODD (5.0%)	IL-1β, IL-6, TNF-α	↑ IL-6 in depression w/and w/o trauma vs HC. ↑ TNF-α in depression vs HC. TNF-α and inhibitory accuracy were inversely correlated in the depression w/trauma sample. IL-6 and IL-1β were not associated with executive function. BMI was positively correlated with IL-6. Quality Rating: High
Lee et al., 2020*	Korea	Clinical (outpatient)	13-18y	K-SADS-PL (DSM-IV)	First-episode MDD Co-morbidity: ADHD (16.0%) OCD (4.0%) Anxiety (4.0%) SoP (4.0%)	IL-1β, IL-6, TNF-α, IL-2, IL-4, IL-10, IFN-γ	↓ IL-2, IFN-γ, TNF-α, and IL-10 in MDD vs HC. ↑ IL-2, IFN-γ, IL-10 in MDD after 12 weeks of SSRI/TCA treatment. Negative correlation of depression scores with IFN-γ and IL-10. Quality Rating: Moderate
Öztürk et al., 2020*	Turkey	Clinical (outpatient)	13-18y	K-SADS-PL (DSM-V)	MDD Co-morbidity: Anxiety (58.3%) ADHD (4.2%)	IL-6, IL-1β, IFN-γ, CRP	No significant difference between MDD vs HC for all cytokines tested. Significant negative correlation between IL-6 and DSM-V level 2 depression and irritability. Significant negative correlation between IFN-γ and Kyn/TRP levels. Quality Rating: High
Study	Country	Participants	Age range	Primary Diagnostic method	Internalizing disorder/co-morbidity	Cytokines investigated	Summary of findings
Perry et al., 2020*	England	Community (birth cohort)	7-18y	CIS-R (ICD-10)	Depression Co-morbidity: Not specified	CRP	No significant difference between depressed and non-depressed for CRP levels at age 18. No significant correlation between CRP and depression severity at age 18. BMI associated with current depression after adjusting for raised CRP longitudinally at age 9 and 18. Quality Rating: Moderate
Reddy et al., 2020*	USA	Clinical (outpatient) and Community	15-18y	MINI v.6 (DSM-V)	MDD Co-morbidity: Not specified	IL-6, TNF-α, IL-8 IL-10	↑ TNF-α in MDD vs HC. Positive correlation between sleep quality and hyperactivity scores and TNF-α.TNF-α indirect link between MDD and sleep disturbance in adolescents. Quality Rating: Moderate
Chen et al., 2021*	Taiwan	Clinical (outpatient)	12-19y	MINI (DSM-V)	First episode MDD Co-morbidity: Not specified	CRP, IL-6, TNF-α, IL-2	No significant difference between MDD vs HC for CRP, IL-6, TNF-α. ↓ IL-2 in MDD vs HC. Quality Rating: Moderate
Gomes et al. (2021)	Brazil	Community (birth cohort)	7-18y	MINI (DSM-V)	MDD, GAD Co-morbidity: Not specified	CRP, IL-6	Diet quality at 18 was associated with lower levels of CRP. No association between CRP or IL-6 and MDD or GAD diagnosis at 18. Quality Rating: Moderate
Study	Country	Participants	Age range	Primary Diagnostic method	Internalizing disorder/co-morbidity	Cytokines investigated	Summary of findings
Liu et al., 2021*	USA	Clinical (outpatient) and Community	12-20y	K-SADS-PL (DSM-IV)	MDD, Anxiety Co-morbidity: Dysthymia (7.9%) DNOS (3.9%) Other mood (4.7%) ODD (9.5%) ADHD (23.6%) OCD (1.6%) Other (2.4%)	CRP	Significant positive correlation between CRP levels and BMI in psychiatric group. No difference in CRP levels between psychiatric group and HC. No correlation between CRP and anxiety, suicidality, or anhedonia symptom measures. Quality Rating: Moderate
Martins et al., 2021*	USA	Clinical (outpatient)	15-20y	DISC-IV (DSM-IV)	MDD Co-morbidity: Not specified	IL-6, IL-8, MCP-1, TNF-α, IL-1β	No difference in tested cytokines between MDD vs HC at baseline or 8-month follow up. Positive correlation between depression scores and MCP-1 at 8-month follow up only. Quality Rating: Moderate
Peters et al., 2021*	USA	Clinical (outpatient) and Community	12-17y	K-SADS (DSM-IV)	Depression Co-morbidity: PD (5.9%) SoP (20.6%) AG (2.9%) GAD (47.1%) PTSD (11.8%) ADHD (14.7%) ODD (5.9%)	IL-1β, IL-6, TNF-α	↑ IL-6 in depression vs HC. IL-6 inversely associated with accuracy and discrimination of angry and neutral faces. Quality Rating: High
Study	Country	Participants	Age range	Primary Diagnostic method	Internalizing disorder/co-morbidity	Cytokines investigated	Summary of findings
Rengasamy et al., 2021b*	USA	Clinical (outpatient) and Community	12-18y	K-SADS-PL f(DSM-IV)	MDD Co-morbidity: Not specified	IL-6, TNF-α	No difference in IL-6 and TNF-α levels in MDD vs HC or medicated vs unmedicated MDD. ↑ TNF-α associated with greater childhood trauma and emotional abuse even after correction for depression severity. Quality Rating: High
Ho et al. (2022)*	USA	Community	13-18y	K-SADS-PL (DSM-IV)	MDD Co-morbidity: Not specified	IL-6, TNF-α	No association between IL-6 and BMI. ↑ IL-6 and TNF-α in MDD vs HC. Quality Rating: High
Li et al., 2022*	China	Clinical (outpatient)	12-20y	K-SADS-PL (DSM-IV)	First-episode MDD Co-morbidity: Not specified	IL-6, TNF-α	↑ IL-6 and TNF-α in first-episode MDD vs HC. Positive correlation between IL-6 and TNF-α in all participants. IL-6 positively correlated with emotional abuse, physical neglect and total trauma. TNF-α positively correlated with total trauma. Quality Rating: Moderate

Note: * = study was included in meta-analysis, y = years, MDD = Major Depressive Disorder, DNOS = Depression Not Otherwise Specified, IL = interleukin, NS = Not Significant, TNF = Tumor Necrosis Factor, IFN = interferon, γ = gamma, α = alpha, HC = Healthy Controls, GAD = Generalized Anxiety Disorder, PD = Panic Disorder, SoP = Social Phobia, SAD = Separation Anxiety Disorder, PTSD = Post-Traumatic Stress Disorder, MVA = Motor Vehicle Accident, SP = Specific Phobia, DYS = Dysthymia, AG = Agoraphobia, GM-CSF = Granulocyte-Macrophage Colony-Stimulating Factor, BD = Bipolar Disorder, OCD = Obsessive Compulsive Disorder, LPS = LipoPolySaccharides, DEX = Dexamethasone, VEGF = Vascular Endothelial Growth Factor, G-CSF = Granulocyte Colony-Stimulating Factor, FGF = Fibroblast Growth Factor, Flt3-L = Fms-like tyrosine kinase 3 ligand, MCP, Other = Other Disorders, ADHD = Attention Deficit Hyperactivity Disorder, SSRI = Selective Serotonin Reuptake Inhibitors, TCA = Tricyclic Antidepressant, K-SADS(-PL) = Kiddie Schedule for Affective Disorders and Schizophrenia(-Present & Lifetime), ICD-10 = International Classification of Diseases (10th revision), DSM = Diagnostic and Statistical Manual of Mental Disorders, ODD = Oppositional Defiant Disorder, SCID-I/NP = Structured Clinical Interview for DSM (non-patient edition), DAWBA = Development and Well-being Assessment, MINI = Mini International Neuropsychiatric Interview, CRP = C-Reactive Protein, Kyn = Kynurenine, TRP = Tryptophan, BMI = Body Mass Index, CIS-R = Clinical Interview Scheduled-Revised.

A wide range of cytokines were examined in these studies: IL-6 (*k* = 23), TNF-α (*k* = 22), IL-1β (*k* = 15), IL-10 (*k* = 10), IFN-γ (*k* = 9), IL-2 (*k* = 7), CRP (*k* = 5), IL-4 (*k* = 5), IL-8 (*k* = 5), IL-17 (*k* = 3), TGF-β (*k* = 2), IL-12 (*k* = 2), IL-5 (*k* = 2), IL-13 (*k* = 1), IL-15 (*k* = 1), and IL-1Ra (*k* = 1). Most of the studies controlled for or analyzed the effect of diurnal variation (e.g., fasting status and/or time of blood collection) (*k* = 23) and medication status (*k* = 22). Cytokine quantification methodologies varied in the included studies: enzyme-linked immunosorbent assay (ELISA) (*k* = 8), sandwich ELISA (*k* = 4), bead-array flow cytometry (*k* = 4), radioimmunoassay (*k* = 1), magnetic-chemiluminescence immunoassay (*k* = 6), sandwich chemiluminescence immunoassay (*k* = 3), and electro-chemiluminescence immunoassay (*k* = 2). Meta-analyses were performed for CRP, TNF-α, IL-6, IL-1β, IL-2, IL-10, and IFN-γ to ensure adequate sample size (at least *k* = 4 studies with available data) for model fitting to reduce false interpretation and given the evidence-base within the existing literature (Himmerich et al., 2019).

### 3.2 Meta-analytic findings

**Fig. 2:**
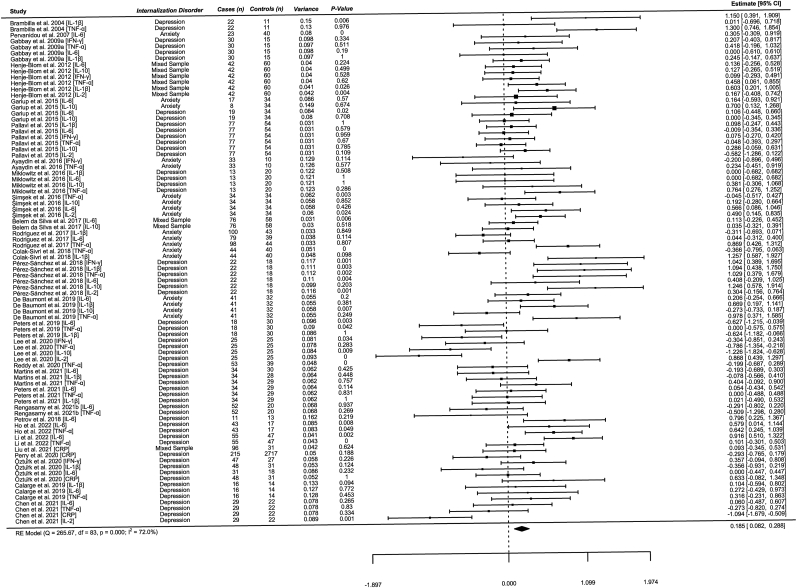
*Note.* Effect sizes are calculated using Hedge's *g*.

Overall, peripheral cytokine levels were observed to be significantly elevated in pediatric internalizing disorders as compared to controls; however, the size of the effect was small (*k* = 84, Hedge's *g* = 0.19, 95% CI [0.08, 0.29], *z* = 3.53, *p* < 0.001). A graphical representation by forest plot is illustrated in Fig. 2. Upon examination of the forest plot, there appears to be significant variation among studies of sample sizes less than 20. An estimated standard deviation of Hedge's *g* (*SD* = 0.44) was calculated to examine the data for outliers that exceeded two standard deviations (−0.69 to 1.07) above or below the calculated Hedge's *g* = 0.19 (Renna et al., 2018). There were five studies identified that could be potential outliers using this method; however, it could be due to small sample size and therefore, they were not removed from the primary analysis due to their low weighting (IL-1β: Brambilla et al., 2004; IL-2: Chen et al., 2021; IL-10: Lee et al., 2020; IFN-γ and TNF-α: Pérez-Sánchez et al., 2018; IL-6: Pervanidou et al., 2007). A sensitivity analysis was performed without the identified outliers (*k* = 78, Hedge's *g* = 0.17, 95% CI [0.08, 0.26], *z* = 3.61, *p* < 0.001), of unmedicated studies only (*k* = 40, Hedge's *g* = 0.26, 95% CI [0.09, 0.44], *z* = 2.91, *p* = 0.004), and with studies that included adolescents only (*k* = 57, Hedge's *g* = 0.14, 95% CI [0.02, 0.26], *z* = 2.36, *p* = 0.018).

### 3.3 Moderator analyses

Moderator analyses were performed on the following variables: cytokine type, percentage of females enrolled, mean BMI, fasting status at blood collection, diagnosis, percentage of sample medicated, and mean age. Data was reported from all the studies for percentage of females enrolled, mean age, and diagnosis, 18 studies reported mean BMI, 22 studies reported the percentage of sample medicated, and 23 studies reported fasting status at blood collection. There was no moderating effect of peripheral cytokine type (IFN-γ (*k* = 7): Hedge's *g* = 0.01, 95% CI [−0.35, 0.38], *z* = 0.07, *p* = 0.947); TNF-α (*k* = 21): Hedge's *g* = 0.21, 95% CI [−0.21, 0.63], *z* = 1.02, *p* = 0.328; IL-6 (*k* = 22): Hedge's *g* = 0.29, 95% CI [−0.12, 0.71], *z* = 1.38, *p* = 0.168; IL-1β (*k* = 14): Hedge's *g* = 0.20, 95% CI [−0.24, 0.65], *z* = 0.90, *p* = 0.367; IL-10 (*k* = 10): Hedge's *g* = 0.06, 95% CI [−0.42, 0.53], *z* = 0.23, *p* = 0.815, CRP (*k* = 4): Hedge's *g* = −0.02, 95% CI [−0.61, 0.56], *z* = −0.08, *p* = 0.937, IL-2 (*k* = 6): Hedge's *g* = 0.08, 95% CI [−0.46, 0.61], *z* = 0.28, *p* = 0.780), percentage of females enrolled (*k* = 83, Hedge's *g* < −0.01, 95% CI [−0.01. 0.00], *z* = −0.79, *p* = 0.430), BMI (*k* = 54, Hedge's *g* = −0.01., 95% CI [−0.08. 0.05], *z* = −0.37, *p* = 0.714), anxiety disorder (*k* = 75, Hedge's *g* = −0.01, 95% CI [−0.27, 0.24], *z* = −0.08, *p* = 0.937), fasting sample (*k* = 67, Hedge's *g* = −0.09, 95% CI [−0.28, 0.10], *z* = −0.93, *p* = 0.354), percentage of sample medicated (*k* = 71, Hedge's *g* = −0.00, 95% CI [−0.01, 0.00], *z* = −1.11, *p* = 0.267), and age (*k* = 84, Hedge's *g* = −0.02, 95% CI [−0.08, 0.04], *p* = 0.543) on the relationship between cytokine levels and pediatric internalizing disorders. There was a significant moderating effect of depressive disorder (*k* = 75, Hedge's *g* = 0.18, 95% CI [0.04, 0.31], *z* = 2.60, *p* = 0.009) and non-fasting status at blood collection (*k* = 67, Hedge's *g* = 0.20, 95% CI [0.06, 0.35], *z* = 2.73, *p* = 0.006) on the relationship between peripheral cytokine levels and pediatric internalizing disorders.

### 3.4 Heterogeneity and publication bias

There was significant between study heterogeneity in the multi-variate analysis including all the examined peripheral cytokines (*Q* (83) = 265.67, *p* < 0.001), when outliers were removed (*Q* (78) = 196.89, *p* < 0.001), unmedicated studies only (*Q* (39) = 167.31, *p* < 0.001), and adolescent studies only (*Q* (59) = 193.32, *p* < 0.001). A high level of variance in heterogeneity for the primary analysis was attributed to the total amount of heterogeneity observed (*I*^*2*^ = 72.0%). It was estimated that 36.0% of the total variance was due to between-cluster heterogeneity, with the remaining 36.0% due to within-cluster heterogeneity. A significant, high amount of heterogeneity was also observed in the moderator peripheral cytokine level analysis (*Q*_*E*_ (77) = 252.11, *p* < 0.001, *I*^*2*^ = 72.6%) and other moderator analyses: percentage of females enrolled (*Q*_*E*_ (81) = 262.32, *p* < 0.001, *I*^*2*^ = 70.7%), fasting status (*Q*_*E*_ (65) = 148.82, *p* < 0.001, *I*^*2*^ = 60.4%), mean BMI (*Q*_*E*_ (52) = 146.17, *p* < 0.001, *I*^*2*^ = 74.9%), mean age (*Q*_*E*_ (82) = 264.74, *p* < 0.001, *I*^*2*^ = 72.3%), percentage of sample medicated (*Q*_*E*_ (69) = 213.99, *p* < 0.001, *I*^*2*^ = 73.3%), and diagnosis (*Q*_*E*_ (73) = 255.24, *p* < 0.001, *I*^*2*^ = NA).

**Fig. 3:**
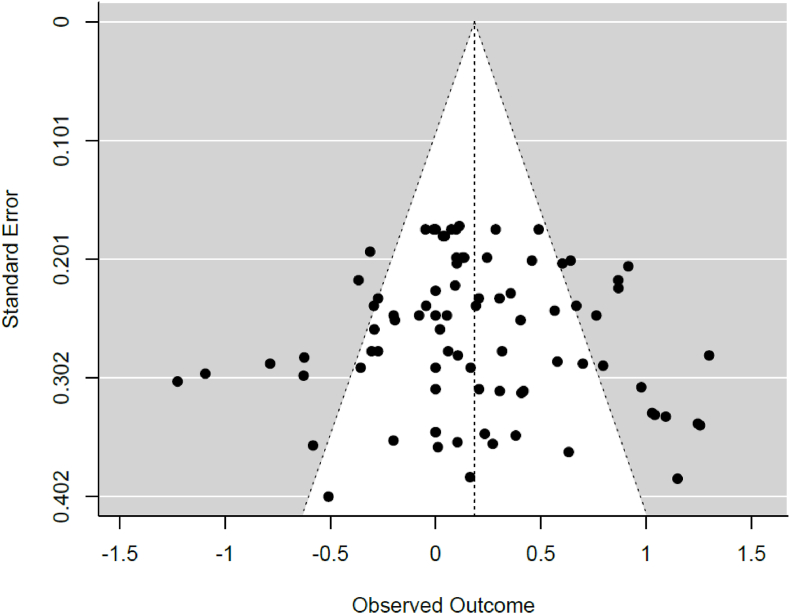
Funnel Plot *Note.* Effect sizes shown on the observed outcome axis are calculated using Hedge's *g*. This plot includes the multi-variate meta-analysis of all the cytokines tested.

Publication bias was evaluated for the statistically significant between-group multi-variate cytokine analysis. A graphical representation of publication bias by funnel plot is illustrated in Fig. 3. There appears to be a trend towards publication bias based on asymmetry of the funnel plot, however publication bias was not observed in the calculated Egger's regression test (*z* = 0.56, *p* = 0.576).

## 4 Discussion

The aim of this systematic review and meta-analysis was to explore the relationship between peripheral cytokines and pediatric internalizing disorders. In this analysis, peripheral cytokine levels were observed to be elevated in pediatric internalizing disorders compared to controls. A recent meta-analysis by Toenders et al. (2022) reported a significant elevation of peripheral IL-1β levels in youth depression (*k* = 7, Cohen's *d* = 0.37) that was not observed in our moderator analysis. However, we acknowledge the differences in our statistical approach (univariate versus multi-variate), larger sample sizes in our IL-1β moderator analysis, and our mixed sample of anxiety and depression as potential reasons for the deviation when comparing our findings. Both of our meta-analyses observed no significant association and high heterogeneity for peripheral IFN-γ, IL-2, IL-6 and TNF-α when comparing youth with psychiatric diagnosis and controls. Our analysis is consistent with the findings of meta-analyses of peripheral cytokines in adults diagnosed with depression (Köhler et al., 2017) and anxiety (Renna et al., 2018). The size of the effect (Hedge's *g* = 0.15–0.19) observed in this pediatric analysis is smaller than reported in adult analyses (Hedge's *g* = 0.39–0.70); however, this may be due to the differences in pooled sample sizes and statistical methodology employed.

Alternatively, it may be that pediatric individuals have reduced allostatic load (O'Connor et al., 2020) and therefore, greater cytokine responsivity to internalizing symptoms that attenuate prior to formal diagnosis (Byrne et al., 2013). Pro-inflammatory peripheral cytokines in youth may be state-dependent and perpetuate symptoms of internalization in the acute phase when exposed to psychosocial or environmental stress (Rohleder and Miller, 2008; Michels et al., 2018). The studies included in this analysis had youth of varying ages and developmental stages. This could be influential on the study findings given that the pubertal stage and increases in sex hormones has been shown to meaningfully influence cytokine levels (Zolin et al., 2015; Mills et al., 2013; Stumper et al., 2020). This is supported by the findings of the sensitivity analysis of adolescent studies only where we observed a reduction in effect size (Hedge's *g* = 0.14). Despite these findings, it remains difficult to ascertain the influence and directionality of the effect of peripheral cytokines in pediatric internalizing disorders due to methodological variation between studies, high heterogeneity statistics, and lower statistical power compared to adult studies.

In adult samples, the most consistent association between cytokines and internalizing psychopathology is elevations in IL-6 (Himmerich et al., 2019). IL-6 is a pro-inflammatory cytokine produced by macrophages involved in the activation of adaptive immune cells and stimulating release of prostaglandins and other cytokines (e.g., IL-10, IL-13, IL-5, IL-7) in response to acute biological and social stress (Chiang et al., 2017). We were unable to find any association with IL-6 inducer, C-reactive protein, and pediatric internalizing disorders, which is consistent with the null findings in the literature (Calarge et al., 2019; Chaiton et al., 2010; Copeland et al., 2012b; Gomes et al., 2021; Liu et al., 2021; Oztürk et al., 2020; Perry et al., 2020). We were also unable to find an association with basal IL-6 levels and pediatric internalizing disorders. This is supported by cross-sectional studies that continue to report equivocal (Gariup et al., 2015; Henje-Blom et al., 2012; Li et al., 2022; Peters et al., 2019, 2021) or null findings in pediatric samples investigating IL-6 (Byrne et al., 2013; Calarge et al., 2019; Freed et al., 2019; Gabbay et al., 2009a, 2009b; Gomes et al., 2021; Oztürk et al., 2020; Petrov et al., 2018; Reddy et al., 2020). However, some evidence has shown an association between depressive symptoms and longitudinal increases in IL-6 throughout early childhood and adolescence (Chu et al., 2019; Kautz et al., 2020). Previous research has identified several variables that are influential in IL-6 levels in children and adolescents that may be responsible for the diversity of findings observed, including disturbed sleep (Fernandez-Mendoza et al., 2017; Toenders et al., 2022; Reddy et al., 2020), adiposity (Chiang et al., 2017; McInnis et al., 2014; Petrov et al., 2018), childhood trauma (Li et al., 2022; Miller et al., 2009; Peters et al., 2019; Russel et al., 2019), executive functioning (Peters et al., 2019; Starr et al., 2016), emotional perception (Peters et al., 2021) and psycho-social stress (Colak Sivri et al. 2018; Flouri et al., 2019; Slavich et al., 2015). Many of the studies included in this meta-analysis did not account for these influential variables and therefore, in this study, we were unable to explore these potential mediating relationships. Anti-depressant medication has been previously reported to influence IL-6 levels (Mills et al., 2013); however, this has not been replicated in recent pharmacological studies in pediatric depression (Lee et al., 2020; Martins et al., 2021; Pérez-Sánchez et al., 2018). A study by Lee et al. (2020) found no significant change in IL-6 levels in a sample of medication naïve, first-episode adolescents with major depressive disorder after 12 weeks of anti-depressant treatment. Another study by Pérez-Sánchez et al. (2018) also found that IL-6 levels decreased after 4 weeks of fluoxetine treatment and normalized to baseline levels after 8 weeks in a sample of adolescents with major depressive disorder. Further study is warranted to determine the mechanism of the mediating role that IL-6 may have in pediatric internalizing disorders.

There is some evidence that TNF-α may play a role in mediating inflammatory activity in pediatric internalizing disorders (Kautz et al., 2020; O'Connor et al., 2020; Rengasamy et al., 2021b). TNF-α is produced by macrophages in the initial inflammatory response against pathogens and cytotoxicity (Peters et al., 2019). It has also been implicated in homeostatic regulation of synaptic plasticity (e.g., learning and memory) (Moriarity et al., 2019), sleep (Reddy et al., 2020), appetite (Himmerich et al., 2019), and body temperature (Mills et al., 2013). It has been speculated that TNF-α may be mediating the link between somatic depressive symptoms and treatment refraction in pediatric internalizing disorders (Amitai et al., 2016, 2020; Toenders et al., 2022). Elevated TNF-α levels have been associated with pediatric obsessive-compulsive disorder (OCD) and major depressive disorder under basal conditions and after lipo-polysaccharide stimulation (Rodríguez et al., 2017). There are also studies that were unable to replicate this association (Freed et al., 2019) or reported reduced levels being associated with major depressive disorder (Lee et al., 2020). In this transdiagnostic meta-analysis, we did not find a significant relationship between pediatric internalizing disorders and TNF-α. Similar to IL-6, TNF-α levels can be modulated by various endogenous (e.g., sex hormones, BMI, cognition) and exogenous (e.g., exercise, diet, smoking) factors (Mills et al., 2013; Peters et al., 2019), which may contribute to the large heterogeneity observed between studies. Furthermore, there is mounting evidence of an association between childhood trauma and TNF-α (Peters et al., 2019; Rengasamy et al., 2021b), which may be a confounder in previous studies with pediatric internalizing disorders. Future research should aim for better sociodemographic, lifestyle, and clinical characterization of the pediatric sample to further understand the role of TNF-α in pediatric internalizing disorders.

IL-2 is an immunologically important cytokine that promotes production of TNF-α and IFN-γ and enhances cytolytic activity of the immune system (Gabbay et al., 2010). Decreased peripheral IL-2 levels has been implicated in internalizing and externalizing symptoms in pediatric and adult samples (Himmerich et al., 2019; Lee et al., 2020). Reduction of IL-2 levels in psychiatric disorders has been hypothesized to be a result of increase HPA axis activation via IDO (indoleamine 2,3-dioxygenase) mediated serotonin depletion and increased noradrenergic activity through the sympathetic nervous system (Gabbay et al., 2010; Himmerich et al., 2019). In pediatric studies, the relationship between IL-2 and internalizing disorders remains unclear. Studies of adolescent first-episode MDD report decreased levels of IL-2 relative to non-psychiatric controls (Lee et al., 2020) whereas studies of anxiety, OCD, and depression report increased levels of IL-2 (Henje-Blom et al., 2012; Pallavi et al., 2015; Pérez-Sánchez et al., 2018; Simsek et al., 2016). It is plausible that increased IL-2 levels may be associated with maintenance of illness severity and decreased IL-2 associated with symptom onset (Chen et al., 2021). However, treatment with SSRI has been associated with increased IL-2 levels and reduced symptoms in pediatric depression and anxiety in some studies (Henje-Blom et al., 2012; Lee et al., 2020; Pallavi et al., 2015; Pérez-Sánchez et al., 2018).

IL-1β, IFN-γ and IL-10 are the remaining three cytokines examined in this meta-analysis. IL-1β promotes recruitment of inflammatory molecules during the acute inflammatory response and is responsible for exacerbating immune responses in chronic illness (Pérez-Sánchez et al., 2018). IL-1β has also been implicated in autoimmunity and immunity related disorders such as OCD (Rodríguez et al., 2017). Few studies have reported an association between elevated IL-1β levels and pediatric internalizing symptom severity (Brambilla et al., 2004; Henje-Blom et al., 2012; Pérez-Sánchez et al., 2018; Rodríguez et al., 2017). A study by Calarge et al. (2019) observed a correlation between IL-1β levels and neurovegetative symptoms in unmedicated adolescents with depression. There is no robust association of IL-1β levels and pediatric internalizing disorders or symptom severity based on review of the literature. IFN-γ inhibits replication of pathogens and virus-infected cells by activating immune system defence cells (T lymphocytes, natural killer (NK) cells, dendritic cells, and macrophages) (Gabbay et al., 2009a). Conflicting findings have been reported for IFN-γ in pediatric samples with elevated levels seen in MDD (Gabbay et al., 2009a; Henje-Blom et al., 2012) and MDD with suicidal behavior (Gabbay et al., 2009b), but also reduced levels in MDD (Lee et al., 2020) and PTSD (Ayaydin et al., 2016). This discrepancy may be due to a strong correlation between IFN-γ levels and severity of psychopathology as these studies have meaningful differences in the severity of their samples (moderate-to-severe inpatients/outpatients vs mild-to-moderate community/outpatient samples). IL-10 is the only anti-inflammatory cytokine examined in this meta-analysis. IL-10 has a vital role in down-regulation of the immune system cells in the acute inflammatory response and has also been associated cell tissue repair and neuroprotection (Gariup et al., 2015). Deficiency in IL-10 has been characterized in many auto-immune and inflammation-associated diseases (Hesse et al., 2011; Huizinga et al., 2000). Interestingly, two studies have reported increased IL-10 in pediatric internalizing disorders compared to controls under basal conditions (Gariup et al., 2015; Henje-Blom et al., 2012). A study by Lee et al. (2020) reported decreased IL-10 in first episode MDD at baseline compared to controls that increased after treatment with anti-depressants. Despite these reports, no relationship between IL-1β, IFN-γ or IL-10 and pediatric internalizing disorders was observed in this meta-analysis.

We did not find any moderating effects of anxiety diagnosis, percentage of females enrolled, mean BMI, mean age, percentage of sample medicated, and fasting blood collection in the relationship between pediatric internalizing disorders and cytokines. The large heterogeneity and low sample size in these analyses may be masking possible mediating relationships with these variables. In this meta-analysis, there was a greater proportion of female participants, and it has been observed that female adolescents have a larger variation in peripheral cytokine levels as compared to males (Dorn et al., 2016; Moriarity et al., 2019). Anti-depressant treatments have produced conflicting results in pharmacological pediatric studies producing increased or decreased peripheral cytokine levels (Amitai et al., 2016, 2020; Lee et al., 2020) and in some cases, no effect after 8 weeks of administration (Becerril-Villanueva et al., 2019; Pérez-Sánchez et al., 2018). Despite the lack of association with BMI, there is evidence for higher levels of adiposity contributing to the relationship between cytokine levels and internalizing symptoms (Byrne et al., 2015; Chiang et al., 2017; Petrov et al., 2018). Age has been identified as an important confounder in immunological research; however, it did not moderate the relationship in our analysis. This may be due to the large age range of participants included (6–20 years old) and differences in pubertal timing and stage as evidenced in our analysis of studies with adolescents only (12–20 years old). A moderating effect was observed in studies with non-fasting samples and therefore, diet quality and lifestyle factors may play an important role in cytokine measurement and observed reduction of peripheral pro-inflammatory cytokine levels (Gomes et al., 2021; Oddy et al., 2018). A second moderating effect was observed in the diagnosis analysis with depression largely driving the relationship between pediatric internalizing disorders and cytokines. This result may further reinforce the accumulating evidence that cytokines are associated with somatic symptoms (Liu et al., 2020) and physical functioning, and less so with cognitive symptoms (Freed et al., 2019). Alternatively, it is also plausible that since an anxiety diagnosis often precedes depression in pediatric samples that cytokine dysregulation plays a more prominent role during symptom recurrence and chronic stages of illness (Jonker et al., 2017; Kiecolt-Glaser et al., 2015). This is supported by recent longitudinal studies that have shown increased levels of IL-6 and TNF-α over the duration of multiple episodes of depressive illness (Chu et al., 2019; Copeland et al., 2012a; Duivis et al., 2015; Kautz et al., 2020; Miller et al., 2009). Further research should attempt to further examine symptom severity longitudinally with multiple time points to determine the trajectory of these cytokines during and between psychopathological episodes.

There are several limitations in this study. This systematic review was not pre-registered, and a protocol was not drafted on PROSPERO in accordance with the PRISMA 2020 guidelines. However, efforts were made to follow the PROSPERO guidelines whenever possible. Peripheral cytokine data was collected cross-sectionally from the included studies and therefore, is only representative of group differences during a specific time-point. It is difficult to draw conclusions about the role of cytokines from this data given that cytokine responsivity is dynamic and intimately involved with other mechanistic stress response systems in the body (Flouri et al., 2020). Future studies should collect multiple stress markers (e.g., cortisol, corticotropin-releasing hormone) to determine relationships between cytokines levels and stress response markers within pediatric internalizing disorders. Group differences were examined based on formal diagnosis only and therefore, the findings may not be representative or generalizable to that of sub-threshold internalization symptoms outside of a clinical setting (Caserta et al., 2011). Symptom severity could not be examined during the moderator analysis due to data constraints, which would have allowed examination of peripheral cytokine relationships with symptom measures as evidenced in recent studies (Chiang et al., 2015; Freed et al., 2019). Future research should attempt to better characterize their sample to include more symptom homogenous groups within pediatric internalizing disorders to better understand potential trans-diagnostic relationships. Serum and plasma measurement of peripheral cytokines is non-specific and therefore, the elevation in cytokines observed in pediatric individuals with internalizing disorders cannot be attributed to a specific organ system or action within the body. The meta-analysis could not overcome the constraints of the inherent variation in the studies included, namely the differences in laboratory methodology, statistical analysis, and sample characterization. Furthermore, we were unable to perform analyses of other cytokines and chemokines underrepresented in pediatric research that have been identified in the literature to be empirically associated with depression (e.g., IL-13, IL-12) or immunologically important (e.g., IL-1, IL-2, IL-4, IL-5, TGF-β, see Himmerich et al., 2019). Lastly, the statistical approach of using a multi-variate random effects model may not adequately represent the effects of individual cytokines and their characterized function within pediatric internalizing disorders.

## 5 Conclusion

This exploratory meta-analysis provides evidence of potential cytokine dysregulation in an inclusive group of pediatric internalizing disorders. The findings suggest that cytokine dysregulation is inconsistent among the pediatric internalizing disorders examined and subject to large variability by endogenous and exogenous factors. Given the limited number of studies in this exploratory meta-analysis, more research is required to disentangle the relationship between peripheral cytokines and pediatric internalizing disorders to understand the role that cytokine dysregulation plays in the etiology and maintenance of symptoms. Future research should examine peripheral cytokines longitudinally to evaluate dynamic changes in peripheral cytokine levels with the emergence or persistence of pediatric depressive symptoms.

## Availability of data, code and other materials

The data analyzed and R scripts can be made available upon request to the corresponding author.

## Funding

None.

## Declaration of competing interest

The authors of this systematic review declare no competing interests.
